# Effects of Wilting and *Lactobacillus plantarum* Addition on the Fermentation Quality and Microbial Community of *Moringa oleifera* Leaf Silage

**DOI:** 10.3389/fmicb.2018.01817

**Published:** 2018-08-06

**Authors:** Yi Wang, Cheng Wang, Wei Zhou, Fu-yu Yang, Xiao-yang Chen, Qing Zhang

**Affiliations:** ^1^College of Forestry and Landscape Architecture, Guangdong Province Research Center of Woody Forage Engineering Technology, Guangdong Research and Development Centre of Modern Agriculture (Woody Forage) Industrial Technology, Guangdong Key Laboratory for Innovative Development and Utilization of Forest Plant Germplasm, State Key Laboratory for Conservation and Utilization of Subtropical Agro-Bioresources, Integrative Microbiology Research Centre, South China Agricultural University, Guangzhou, China; ^2^College of Animal Science and Technology, China Agricultural University, Beijing, China

**Keywords:** *Moringa oleifera* Lam., *Lactobacillus plantarum*, silage, fermentation quality, bacterial community

## Abstract

The objective of this study was to evaluate the effects of wilting and *Lactobacillus plantarum* (LP) addition on the silage fermentation quality and microbial community of *Moringa oleifera* Lam. leaf silage. Unwilted (direct-cut) or wilted *M. oleifera* leaves were prepared either with or without LP (1.0 × 10^6^ cfu/g) followed by either 60 or 120 days of ensiling, leading to eight treatment groups. The results showed that lactic acid was the dominant fermentation product, and no butyric acid was detected for any of the treatments. Higher acetic acid and propionic acid were detected during the fermentation of wilted silage compared to unwilted silage. Although NH_3_-N content increased after wilting, the content was far below 10% of the dry matter (DM). In addition, higher pH was observed after 120 days of ensiling compared to 60 days. Wilting also influenced the bacterial community structure. *Lactobacillus* was the most dominant genus in unwilted samples while *Enterobacteriales*, *Weissella*, and *Pantoea* were the most dominant genera in wilted samples. Furthermore, the relative abundance of undesirable microorganisms was far below that of lactic acid bacteria in all treatments. In summary, wilting had significant effects on fermentation quality, and it was shown that *M. oleifera* leaves can undergo quality ensiling directly without the addition of LP.

## Introduction

With the rapid development of the economy in China, the availability and price of concentrates, particularly regarding adequate and high protein sources, have become serious problems for animal feed. Many fodder trees with higher levels of crude protein, digestible nutrients, and minerals have been used to ameliorate this problem (Alsersy et al., 2015; Kholif et al., 2016). One of these fodder trees is *Moringa oleifera* Lam., which has received considerable attention during recent years (Zheng et al., 2016). *M. oleifera* is commonly known as the “drumstick tree.” It has a high biomass yield and is widely distributed almost worldwide (Soliva et al., 2005). The leaves are rich in protein (with an adequate amino acid profile), vitamins, essential fatty acids, and a rare combination of bioactive secondary metabolites (flavonoids and chlorogenic acid); the leaves can fulfill the recommended daily nutrient requirements for ruminants, especially goats and sheep (Adegun and Aye, 2011; Nouman et al., 2014; Babiker et al., 2017; Elghandour et al., 2017). Therefore, *M. oleifera* may be a potential feed source. However, because of the large variation in feed production over the year, there is a need to develop a method to preserve the plant for use when high-quality feed sources are scarce.

Ensiling is a common way to preserve forage crop, which can prolong the maximum storage duration and improve palatability. During the ensiling process, water-soluble carbohydrates (WSC) are converted into lactic acid by lactic acid bacteria (LAB) under anaerobic conditions, and thus the pH drops to a certain extent (to approximately 4.0–4.5) (Herrmann et al., 2011; Li and Nishino, 2011). Additives can be used to improve the ensiling process. Many types of homo-fermentative LAB, such as *Lactobacillus plantarum* (LP), *Enterococcus faecium*, and *Pediococcus* spp., have been proposed as effective stimulants to reinforce lactic acid fermentation (Tian et al., 2017; Zheng et al., 2017). During ensiling, the moisture contents of material may affect the silage fermentation quality. Many researchers have concluded that wilting is an effective technique for suppressing the growth of undesirable bacteria in silage (Haigh and Chapple, 1998; Cazzato et al., 2011; Liu et al., 2011; Nishino et al., 2012).

Over the last few years, the basic preservation form of *M. oleifera* leaves has been leaf meal, and little research has been carried out on silage modulation technology. The purpose of the current study was to investigate the influence of wilting and LP addition on the fermentation characteristics and bacterial community of *M. oleifera* leaf silage and to provide more-detailed information about *M. oleifera* for ensiling.

## Materials and Methods

### Forage Harvest and Silage Preparation

*Moringa oleifera* leaves were cultivated and harvested on July 20, 2017, from an experimental field of South China Agricultural University (23.24°N, 113.64°E). Pre-ensiled samples were analyzed along with samples from eight treatments group, which involved different combinations of LP addition or control (no LP addition), wilting or no wilting, and ensiling for 60 or 120 days.

The unwilted material had a dry matter (DM) content of 250 g/kg, while the wilted material was wilted for about 12 h to obtain a target DM content of 400 g/kg. The material was then chopped using a crop chopper into sections of approximately 2 cm in length. LP was isolated from *Leymus chinensis* silage and was identified according to Zhang et al. (2016a). The final application rate was 1.0 × 10^6^ colony-forming units (cfu)/g of fresh material (FM) (Chen et al., 2016). About 170 g pre-ensiled sample was packed into a plastic bag (20 × 30 cm; Dongguan Bojia Packaging Co. Ltd, Dongguan, China) using a vacuum sealer (Lvye DZ280; Dongguan Yijian Packaging Machinery Co., Ltd., Dongguan, China). Ensiling for 60 and 120 days was performed in triplicate, which involved storing the material at room temperature.

### Analysis of Microbial Population, Organic Acid, and Chemical Composition

Immediately after the bags were opened, the samples (20 g) were blended with 180 ml sterilized saline solution (8.5 g/L NaCl), and serially diluted from 10^-1^ to 10^-6^. The number of LAB growing on de Man, Rogosa, Sharpe (MRS) agar incubated at 30°C for 48 h under anaerobic conditions (LRH-250, Shanghai, China) was counted. Yeast was counted on Rose Bengal Agar, incubated at 28°C for 48 h. Coliform bacteria was counted on Violet Red Bile Agar incubated at 30°C for 48 h under aerobic conditions. The colony counts represented the numbers of viable microorganisms in cfu/g FM.

Next, 20 g of each silage sample was homogenized in a blender with 180 mL distilled water for 1 min and then filtered through four layers of cheesecloth and filter paper. The pH and concentration of ammonia nitrogen (NH_3_–N) in the filtrate were then determined. The pH was measured immediately with a glass electrode pH meter (PHS-3C; INESA Scientific Instrument Co. Ltd, Shanghai, China). Concentrations of organic acid were measured using high-performance liquid chromatography (HPLC). The HPLC conditions were as follows: column, Shodex RSpak KC-811S-DVB gel C (8.0 mm × 30 cm; Shimadzu, Tokyo, Japan); oven temperature, 50°C; mobile phase, 3 mmol/L HClO_4_; flowrate, 1.0 mL/min; injection volume, 5 μL; and detector, SPD-M10AVP (Tian et al., 2017). The concentration of NH_3_–N was analyzed using the method of Broderick and Kang (1980).

DM content was determined by oven drying at 65°C for 48 h. Crude protein was analyzed according to the methods of the Association of Official Analytical Chemists (AOAC, 1990). Neutral detergent fiber, acid detergent fiber, and acid detergent lignin contents were measured according to the procedures of Van Soest et al. (1991). WSC content was determined using the anthrone method (Murphy, 1958).

### Microbial DNA Isolation and PCR Amplification

For the molecular analysis of the microbial community composition after ensiling, genomic DNA was extracted from silage samples using an E.Z.N.A. stool DNA Kit (Omega Biotek, Norcross, GA, United States) according to the manufacturer’s protocols. Primers 341F (CCTACGGGNGGCWGCAG) and 806R (GGACTACHVGGGTATCTAAT) were used to amplify the 16S rDNA V3-V4 region of the eukaryotic ribosomal RNA gene. The steps included prior denaturation at 95°C for 2 min, followed by 27 cycles of denaturation at 98°C for 10 s, annealing at 62°C for 30 s, elongation at 68°C for 30 s, and a final extension at 68°C for 10 min. PCR reactions were performed using 50 μL reaction mixture containing 5 μL of 10 × *Thermococcus kodakaraensis* (KOD) buffer, 5 μL of 2.5 mM dNTPs, 1.5 μL of each primer (5 μM), 1 μL of KOD polymerase and 100 ng of template DNA. To minimize PCR deviation, for each sample, PCR reactions were conducted in triplicate, and the mixtures of each set of three PCR products were used to perform sequencing.

### Illumina Hiseq2500 Sequencing and Microbial Diversity Analysis

Amplicons were extracted from 2% agarose gels and purified using an AxyPrep DNA Gel Extraction Kit (Axygen Biosciences, Union City, CA, United States) according to the manufacturer’s instructions and quantified using QuantiFluor-ST (Promega, United States). Purified amplicons were pooled in equimolar amounts and paired-end sequenced (2 × 250) on an Illumina platform according to the standard protocols. To obtain high-quality clean reads, any sequences that contained >10% of unknown nucleotides (N) and <80% of bases with quality (*Q*-value) >20 were removed. Paired-end clean reads were merged as raw tags using FLSAH (v 1.2.11) with a minimum overlap of 10 bp and mismatch error rates of 2%. Noisy sequences of raw tags were filtered using the QIIME (v 1.9.1) pipeline under specific filtering conditions to obtain high-quality clean tags. The effective tags were clustered into operational taxonomic units (OTUs) at 97% similarity using the UPARSE pipeline. Taxonomic classification at the phylum and genus levels was performed using the ribosome database project (RDP) classifier. The alpha-diversity of samples, mainly regarding the Shannon index, Chao richness estimator, and Good’s coverage estimator, were calculated at 97% identity in QIIME. A principle component analysis (PCA) was conducted, and PCA graphs were plotted in R Statistics.

### Statistical Analysis

Unless otherwise stated, the statistical analyses were conducted using SAS 9.3 software. The significance of the differences between the treatment groups were estimated using Duncan’s multiple range tests. The level of significance and high significance were set to *P* < 0.05 and *P* < 0.01, respectively. GraphPad Prism 5 was used to draw the figures.

## Results

### Chemical and Microbial Characteristics of Pre-ensiled *M. oleifera*

The chemical and microbial compositions of *M. oleifera* leaves before ensiling are shown in **Table 1**. The DM content was 248 g/kg FM, and the WSC content was 100.72 g/kg DM. The LAB count was 4.63 log cfu/g FM. Additionally, the coliform bacteria and yeast counts were 4.81 and 2.30 log cfu/g FM, respectively.

**Table 1:** Chemical and microbial compositions in the pre-ensiled samples.

Items	*M. oleifera* Leaves
DM (g/kg)	248.87
Crude protein (g/kg DM)	150.56
Neutral detergent fiber (g/kg DM)	310.24
Acid detergent fiber (g/kg DM)	210.23
Acid detergent lignin (g/kg DM)	300.80
Water-soluble carbohydrates (g/kg DM)	100.72
Lactic acid bacteria (log cfu/g FM)	4.63
Coliform bacteria (log cfu/g FM)	4.81
Yeast (log cfu/g FM)	2.30

*DM, dry matter; FM, fresh matter; cfu, colony forming units.*

### Chemical and Microbial Characteristics, and Fermentation Quality, of *M. oleifera* Silage

**Table 2** shows the chemical composition, fermentation quality, and microbial population of *M. oleifera* leaf silage. Overall, lactic acid was the dominant fermentation product, whereas, for all treatments, butyric acid, and the coliform bacteria count were below the detectable levels after ensiling. The acetic acid, propionic acid, and NH_3_-N contents were highly significantly increased by wilting (*P* < 0.01). However, the numbers of LAB and yeast were significantly decreased by wilting. Wilting had no significant effect on the lactic acid content or the pH, but the interactions between wilting and number of ensiling days, and between wilting and LP addition, were significant (*P* < 0.05). The number of ensiling days had significant effects on the lactic acid content and the pH, but no significant effect on the acetic acid, propionic acid, and NH_3_-N contents, or on the LAB and yeast counts. Ensiling for 120 days led to a higher pH and lower lactic acid content than ensiling for 60 days. Although LP had a significant effect on NH_3_-N content, the NH_3_-N content varied in a very narrow range. In addition, the wilted samples with LP had lower pH compared with the wilted control samples (without LP). In general, wilting had a significant effect on the neutral detergent fiber and acid detergent fiber contents, but no significant effect on the crude protein content. LP had no significant effect on crude protein, neutral detergent fiber, or acid detergent fiber contents. Higher crude protein contents were obtained after 120 days of ensiling, compared with the same samples after 60 days of ensiling.

**Table 2:** Chemical compositions and fermentation characteristics of wilted *M. oleifera* leaves silage prepared with and without LP and ensiling for 60 and 120 days.

	60 days				120 days				ANOVA					
Items	Unwilted		Wilted		Unwilted		Wilted		W	D	LP	W^∗^D	W^∗^LP	D^∗^LP
	CK	LP	CK	LP	CK	LP	CK	LP						
Lactic acid (g/kg DM)	125.18^a^	77.74^b^	104.66^a^	97.95^a^	61.89^a^	69.16^a^	108.12^a^	93.93^a^	NS	^∗^	NS	^∗^	NS	NS
Acetic acid (g/kg DM)	1.33^a^	0.40^a^	9.19^a^	7.20^a^	0.00^a^	1.33^a^	10.94^a^	8.24^a^	^∗∗^	NS	NS	NS	NS	NS
Propionic acid (g/kg DM)	9.05^a^	2.73^b^	52.73^a^	57.25^a^	5.46^a^	6.68^a^	58.98^a^	56.82^a^	^∗∗^	NS	NS	NS	NS	NS
Butyric acid (g/kg DM)	ND	ND	ND	ND	ND	ND	ND	ND	–	–	–	–	–	–
pH	3.82^a^	3.82^a^	3.93^a^	3.76^b^	3.99^a^	4.08^a^	4.06^a^	3.91^b^	NS	^∗∗^	NS	NS	^∗^	NS
NH_3_-N (g/kg FM)	0.02^a^	0.01^a^	0.14^a^	0.11^a^	0.02^a^	0.03^a^	0.13^a^	0.11^a^	^∗∗^	NS	^∗^	NS	^∗^	NS
Lactic acid bacteria (log cfu/g FM)	5.03^a^	6.83^a^	<2.0^b^	5.3^a^	6.50^a^	6.55^a^	5.21^a^	4.53^a^	^∗∗^	NS	NS	NS	^∗^	NS
Yeasts (log cfu/g FM)	4.8^a^	5.24^a^	3.15^a^	3.70^a^	4.41^a^	3.87^a^	3.96^a^	3.40^a^	^∗∗^	NS	NS	^∗∗^	NS	^∗^
Coliform (log cfu/g FM)	<2.0	<2.0	<2.0	<2.0	<2.0	<2.0	<2.0	<2.0	–	–	–	–	–	–
Crude protein (g/kg DM)	173.81^a^	179.83^a^	185.26^a^	185.3^a^	191.18^a^	191.47^a^	188.87^a^	187.60^a^	NS	^∗∗^	NS	^∗^	NS	NS
Neutral detergent fiber (g/kg DM)	303.08^a^	289.16^a^	298.67^a^	301.62^a^	267.84^a^	280.95^a^	304.45^a^	305.87^a^	^∗^	NS	NS	NS	NS	NS
Acid detergent fiber (g/kg DM)	208.90^a^	190.58^a^	219.57^a^	218.93^a^	178.00^a^	171.99^a^	212.24^a^	223.71^a^	^∗∗^	NS	NS	NS	NS	NS

*“ND”, not detected; “—”, not analyzed; values within the same row under same ensiling days with different superscripts in lowercase letter differ significantly from each other at P < 0.05 ^∗^ and ^∗∗^, significant at P < 0.05 and 0.01, respectively; ‘NS’, no significant; unwilted, direct-cut; wilted, wilted for 12 h. CK, control (no LP addition); LP, Lactobacillus plantarum; W, wilting; D, ensiling days; W^∗^D, the interaction between wilting and ensiling days; W^∗^LP, the interaction between wilting and LP; D^∗^LP, the interaction between ensiling days and LP.*

### Effect of LP and Wilting on Bacterial Community After 60 and 120 Days of Ensiling

Bacterial alpha diversity values are shown in **Table 3**. The recovered reads of the samples ranged from 91855 to 116342, and a total of 2193 OTUs were clustered at a 3% dissimilarity level. The samples with LP had fewer OTUs after 60 days of ensiling, but more OTUs after 120 days of ensiling. The Good’s coverage values of all treatments were around 0.99, revealing that most of the bacteria were detected. Another bacterial community richness estimator, Chao, showed a similar trend to the number of OTUs. The Shannon index of bacterial diversity varied from 2.63 to 2.79 for 60 days of ensiling and from 2.56 to 2.75 for 120 days.

**Table 3:** Alpha diversity of bacterial diversity at the day 60 and 120 days of ensiling.

			Reads	OTU	Chao1	Shannon	Good’s coverage
		M	117,135	118	172	0.47	0.99
	Unwilted	CK	91,855	272	382	2.79	0.99
60 days		LP	100,200	258	337	2.7	0.99
	Wilted	CK	96,347	269	388	2.75	0.99
		LP	99,412	230	303	2.63	0.99
	Unwilted	CK	96,913	248	359	2.74	0.99
120 days		LP	105,162	277	354	2.75	0.99
	Wilted	CK	108,775	259	349	2.58	0.99
		LP	116,342	262	339	2.56	0.99

*M, pre-ensiled sample; unwilted, direct-cut; wilted, wilted for 12 h. CK, control (no LP addition); LP, Lactobacillus plantarum.*

**FIGURE 1:**
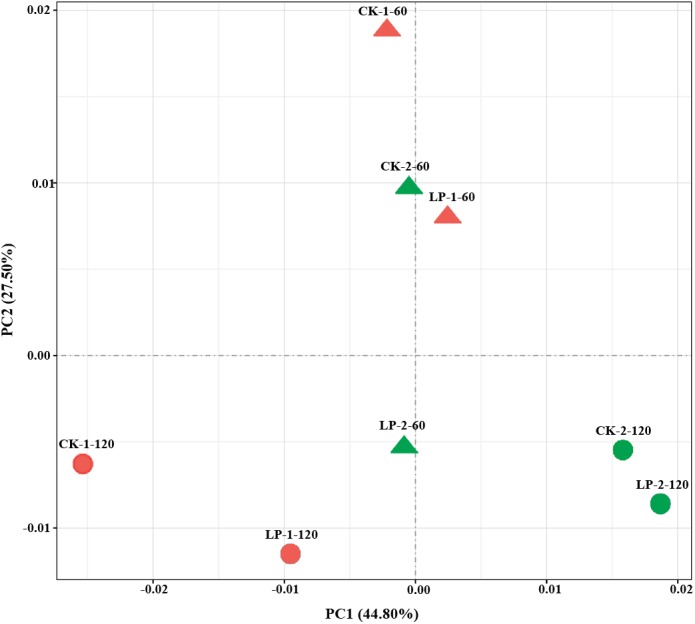
Principle component analysis (PCA) of samples. PC1, principle component 1; PC2, principle component 2; CK, control (no LP addition); LP, *Lactobacillus plantarum*; 1, the material was directed cut; 2, the material was wilted for 12 h; 60, ensiled for 60 days; 120, ensiled for 120 days; CK-1-60, control was directed cut and ensiled for 60 days, the same with other groups.

After ensiling using different treatments, the variance of the bacterial community structure was demonstrated by PCA. As shown in **Figure 1**, principle component 1 (PC1) and 2 (PC2) explained 27.5% and 44.8% of the total variance, respectively. The wilted samples could be separated from the unwilted samples, and the samples ensiled for 60 days could be separated from the samples ensiled for 120 days. However, the samples with LP added had similar bacterial community structure to the control samples after ensiling.

**FIGURE 2:**
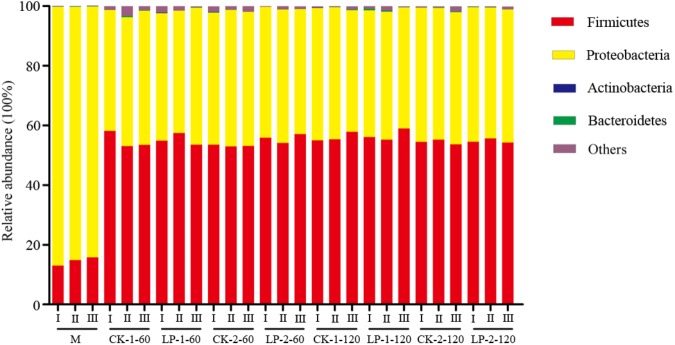
Relative abundance of bacterial at the phylum. M, Pre-ensiled sample; CK, control (no LP addition); LP, *Lactobacillus plantarum*; 1, the material was directed cut; 2, the material was wilted for 12 h; 60, ensiled for 60 days; 120, ensiled for 120 days; CK-1-60, control was directed cut and ensiled for 60 days, the same as other groups; I, II, III, triplicate per treatment.

**FIGURE 3:**
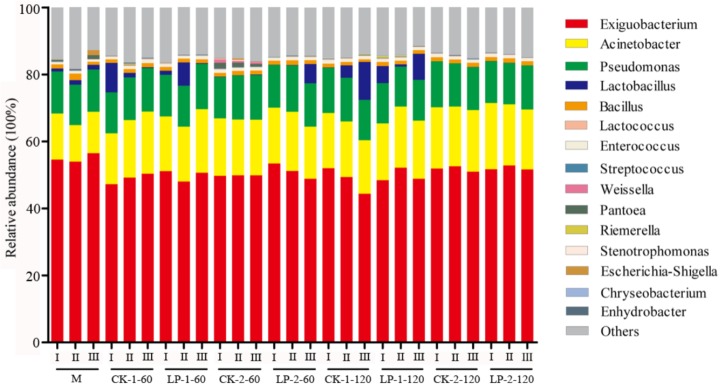
Relative abundance of bacterial at the genus. M, Pre-ensiled sample; CK, control (no LP addition); LP, *Lactobacillus plantarum*; 1, the material was directed cut; 2, the material was wilted for 12 h; 60, ensiled for 60 days; 120, ensiled for 120 days; CK-1-60, control was directed cut and ensiled for 60 days, the same as other groups; I, II, III, triplicate per treatment.

The bacterial community structure of pre-ensiled and silage samples at the phylum and genus levels are shown in **Figures 2**, **3**. As shown in **Figure 2**, the bacterial community was similar at the phylum level, with all groups containing *Proteobacteria*, *Firmicutes*, *Bacteroidetes*, and *Actinobacteria*. Among these, *Proteobacteria* and *Firmicutes* were the most abundant bacteria in the pre-ensiled samples, the relative abundance of which was 84.11–86.85% and 12.99–15.80% (**Supplementary Table S1**), respectively. However, after ensiling, the relative abundance of *Proteobacteria* decreased, while *Firmicutes* increased, accounting for 40.58–45.88% and 52.99–58.97%, respectively.

Regarding genera, *Exiguobacterium*, *Acinetobacter*, and *Pseudomonas* were identified as the three most dominant genera in the pre-ensiled samples. They were also detected after 60 and 120 days of ensiling. However, the relative abundance of *Exiguobacterium* decreased slightly. After ensiling, the major desirable LAB were *Lactobacillus* (0.01–11.29%), *Enterococcus* (0.61–1.1%), *Lactococcus* (0.07–0.26%), *Streptococcus* (0–0.27%), and *Weissella* (0–1.02%) (**Supplementary Table S2**), but the relative abundance associated with each treatment was low. Wilting influenced the relative abundance of *Lactobacillus* and *Weissella.* After 60 days of ensiling, the abundance of *Lactobacillus* was decreased by wilting from 0.28–8.23% to 0.05–0.15%. In contrast, the abundance of *Weissella* was increased by wilting from 0.003–0.007% to 0.51–1.02%.

**FIGURE 4:**
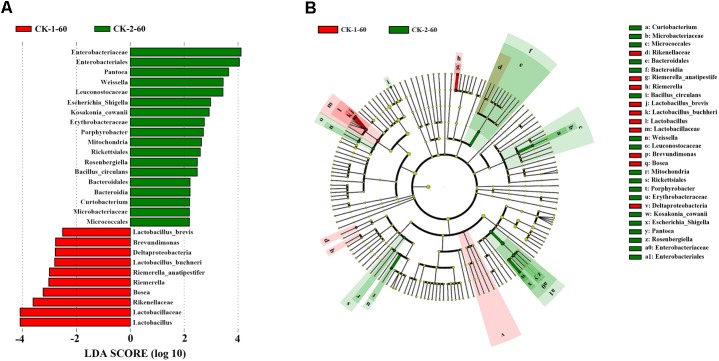
Comparison of microbial variations between CK-1-60 and CK-2-60, using the LEfSe online tool. **(A)** Histogram of the LDA scores for differentially abundant features between CK-1-60 and CK-2-60. The threshold on the logarithmic LDA score for discriminative features was set to 2.0. **(B)** Cladogram for taxonomic representation of signi?cantly differences. Differences are represented in the color of the most abundant taxa.

The linear discriminant analysis (LDA) effect size (LEfSe) method can be used to analyze the differences in bacterial flora between groups and identify the specific bacterial flora in each group (LDA score > 2.0). As shown in **Figure 4**, overall, wilting had a significant effect on the bacterial flora. *Lactobacillus*, *Riemerella*, *Bosea*, and *Brevundimonas*, which were most abundant in unwilted silage, and *Enterobacteriales*, *Weissella*, *Pantoea*, *Curtobacterium*, and *Porphyrobacter*, which were most abundant in wilted silage, were the dominant genera that contributed to the differences between wilted and unwilted silage.

## Discussion

Generally, LAB are the main microorganisms that affect silage fermentation by producing the organic acids responsible for its preservation; good preservation of silage requires that the LAB count reaches >10^5^ cfu/g FM (Cai et al., 1998). In the present study, the LAB count in the pre-ensiled samples was relatively low (<5.00 log cfu/g FM), while the undesirable yeast and coliform bacteria counts were relatively high, indicating that *M. oleifera* leaf silage may needs to be controlled by LAB additives for good fermentation.

In addition, it is well known that sufficient WSC content is a crucial factor for silage fermentation, and a WSC content of 60–80 g/kg is adequate for assuring acceptable fermentation quality (Woolford, 1984). The WSC content in the present study was 100.72 g/kg DM, which is even higher than the WSC content of whole crop corn (Zhang et al., 2017). Thus, the WSC content in the present was adequate for good preservation of *M. oleifera* leaves.

The moisture content of material has an important influence on silage fermentation. Low moisture content concentrate WSC, promote lactic acid fermentation, and lead to the production of less NH_3_-N (Broderick et al., 2000). In this study, the microbial population and the organic acid content (particularly the acetic acid and propionic acid contents) were significantly influenced by wilting. Moreover, the high level of acetic acid and propionic acid in wilted silage may be important for aerobic stability (Zhang et al., 2016b); this issue requires further research. Jacxsens et al. (2003) reported that *Pantoea agglomerans* can produce acetic acid, propionic acid, and succinate, and these *Enterobacteriaceae* are able to ferment sugars to acids under anaerobic conditions. The relative abundance of *Enterobacteriaceae* (especially *Pantoea*) was relatively high in wilted silage in the present experiment (**Figure 4**), which may explain the increases in both organic acids.

Lactic acid is the main organic acid underlying the pH reduction observed in ensiled fresh material. McDonald et al. (1991) highlighted that pH is an important indicator of the extent of fermentation and silage quality. Good silage should have a pH ≤ 4.2 and an NH_3_-N content <10% DM. After ensiling, all treatments led to relatively low pH and NH_3_-N content, indicating that the silages were well-preserved regardless of LP addition. Moreover, lactic acid was the dominant fermentation product. This may be due to *M. oleifera* leaves having a high WSC content, which makes them suitable for lactic acid production, like whole crop corn (Kleinschmit et al., 2005). On the other hand, *M. oleifera* leaves have been identified as having high antimicrobial activity (Jayawardana et al., 2015), which might inhibit the growth of LP. In the present experiment, the lactic acid content decreased and the pH increased significantly with prolonged ensiling. This might have occurred because *Lactobacillus* can metabolize lactic acid under sugar-deficient conditions (Lindgren et al., 1900).

Next-generation sequencing can provide a large amount of data for exploring both the taxonomic classifications and activities of silage microbiota (Ni et al., 2017a). However, there are no studies concerning the bacterial community changes involved in *M. oleifera* leaf ensiling. In the present study, the alpha diversity values showed that the diversity of the bacterial community was richer after ensiling, which was in accordance with the results of a previous report (Ni et al., 2017b; Zhao et al., 2017). St-Pierre and Wright (2014) found that the phyla *Firmicutes*, *Bacteroidetes*, *Chloroflexi*, and *Proteobacteria* were dominant phyla, and they played important roles in hydrolysis and acidogenesis. Among these phyla, *Firmicutes* is the predominant phylum in most grass silages (Eikmeyer et al., 2013). In the present study, *Proteobacteria* was the most abundant bacteria in the pre-ensiled samples. However, the relative abundance of *Firmicutes* increased after ensiling and it became the dominant phylum. Bao et al. (2016) also reported a similar result regarding the legume *Medicago*. Furthermore, in the present study, the major desirable LAB after ensiling were *Lactobacillus*, *Enterococcus*, *Lactococcus*, *Streptococcus*, and *Weissella*, which play major roles in the inhibition of *Enterobacteria* growth and have also been detected in other materials such as Italian ryegrass and switchgrass (Li and Nishino, 2011; Zhao et al., 2017). The phylum *Proteobacteria* was also an influential component of bacterial community, specifically the genera *Enterobacteria* and *Pantoea*, which can use lactic acid and thus cause nutrient loss (Ridwan et al., 2015). However, the relative abundance of undesirable *Enterobacteriaceae* and *Pantoea* were far below the relative abundance of LAB in all treatments. These results may explain the relatively good fermentation quality of *M. oleifera* leaves. In addition to LAB, large amounts of other bacteria were also observed in *M. oleifera* leaf silage. Among them, *Exiguobacterium* was the dominant genus, and the relative abundance was almost 51.58–66%. Lund and Schleifer (1983) previously reported that *Exiguobacterium* is a Gram-positive facultative anaerobe that is catalase positive and oxidase negative and can ferment glucose to lactic acid, acetic acid, and formic acid. Our result regarding *Exiguobacterium* was quite different from the results of previous studies that reported that *Lactobacillus* and *Pediococcus* became the dominant genera when silage fermentation finished (Bao et al., 2016; Ni et al., 2017b). Therefore, the role of *Exiguobacterium* in the silage fermentation needs further study.

## Conclusion

The silage quality of *M. oleifera* leaves can be ensured by ensiling, regardless of LP addition. All treatments led to a relatively low pH, low NH_3_-N content, and high lactic acid concentration. Wilting significantly increase the acetic acid, propionic acid, and NH_3_-N contents. In addition, the lactic acid content decreased and the pH increased with prolonged silage time. Wilting also changed the bacterial community. *Lactobacillus* was the main dominant genus in unwilted samples, while *Enterobacteriales*, *Weissella*, and *Pantoea* were the main genera in wilted samples.

## Author Contributions

QZ and X-YC formulated and designed the experiments. YW and CW performed the experiments. YW was mainly responsible for analyzing the data and writing the manuscript. WZ and F-YY were involved in the revision of the manuscript.

## Conflict of Interest Statement

The authors declare that the research was conducted in the absence of any commercial or financial relationships that could be construed as a potential conflict of interest.
